# Neovascular Glaucoma: A Rare Presenting Feature of Vogt-Koyanagi-Harada Syndrome

**DOI:** 10.7759/cureus.63753

**Published:** 2024-07-03

**Authors:** Puja Hingorani-Bang, Meghana Kandi, Vandana A Iyer, Shraddha Pawar, Rajesh Pattebahadur

**Affiliations:** 1 Ophthalmology, All India Institute of Medical Sciences, Nagpur, IND

**Keywords:** iris nodules, complicated cataract, poliosis, azathioprine, intravenous methyl prednisolone, granulomatous uveitis, hyphaema, neovascular glaucoma, vogt koyanagi harada

## Abstract

Vogt-Koyanagi-Harada syndrome (VKH) is an uncommon multi-system autoimmune inflammatory disorder characterized by bilateral granulomatous panuveitis with serous retinal detachment accompanied by neurological, auditory, and cutaneous manifestations like headache, hearing loss, vitiligo, and poliosis. It has a female preponderance, usually in middle age. We report the case of a 20-year-old male who presented to us with rapidly progressive visual loss accompanying granular panuveitis, complicated cataract, and a mixed mechanism neovascular glaucoma with acute angle closure. He was treated for IOP control and underwent aggressive immunosuppression and, later, bilateral laser iridotomies. It wasn't until one month after the initial presentation that he presented with vitiligo and poliosis of the eyebrows and eyelashes, clinching the diagnosis of VKH syndrome. This case highlights the diagnostic challenge faced due to acute neovascular glaucoma being the initial presenting feature of VKH; hitherto not documented before, although acute angle closure glaucoma or crisis has occasionally been reported at presentation; the classical VKH presentation being an acute posterior segment uveitis or less commonly, a chronic, recurrent panuveitis presenting with/ without complications. This case underlines the importance of considering VKH syndrome in a patient with bilateral granulomatous panuveitis, as dermatological involvement can emerge later in the disease course, by which time vision might have already been compromised significantly.

## Introduction

Vogt-Koyanagi-Harada syndrome (VKH) is an uncommon multi-system inflammatory disorder characterized by bilateral, granulomatous panuveitis with serous retinal detachment along with neurological, auditory, and cutaneous manifestations like headache, hearing loss, tinnitus, vitiligo, and poliosis.

The inflammation is likely to have an autoimmune aetiology which is directed against melanocytes. Hence, manifestations are race-dependant [1]. In Japan and Singapore, VKH constitutes between 6.8-9.2% of all uveitis, 1.2% in the Middle East, 15.9% in China, 2.2% in India, and between 1-4% in the USA [2]. Non-necrotizing granulomas with late involvement of choriocapillaries and the formation of Dalen-Fuchs' nodules are pathognomonic on histopathology.

The revised diagnostic criteria for Vogt-Koyanagi-Harada syndrome are as follows: [3]

No h/o penetrating ocular trauma or surgery preceding the initial onset of uveitis. 

No clinical or laboratory evidence is suggestive of other disease entities. 

Bilateral ocular involvement* 

Neurological/auditory findings.

Integumentary features.

*with diffuse choroiditis as seen in the early phase: 

-anterior granulomatous uveitis, vitritis, serous retinal detachment, sub-retinal fluid or 

-diffuse choroidal thickening on USG and leaks/ pooling/ staining on fundus fluorescein angiography (FFA)

OR

*with late manifestations, including chronic anterior uveitis or posterior chorioretinal involvement.

The typical VKH presentation is posterior segment uveitis (diffuse choroiditis with serous retinal detachment and disc edema) that may be followed by anterior segment involvement or panuveitis without proper treatment. Acute angle closure glaucoma or crisis may occasionally be found at presentation [2].

The prognosis in a patient aggressively treated with steroids and immunomodulators is fair, with 60% maintaining a visual acuity of 20/30 or better. Chronicity and recurrences may predispose the patients to develop complications like cataracts, glaucoma, and sub-retinal neovascular membrane (SRNVM) that may need to be managed medically, surgically, or with lasers. Initial aggressive treatment may be protective against the development of complications and hence improve visual prognosis [1].

This report discusses the case of a patient whose diagnosis of VKH was challenging owing to the rare initial presentation with neovascular glaucoma accompanying granulomatous panuveitis and made further difficult by the inability to visualize the fundus due to a secondary, complicated cataract.

## Case presentation

A 20-year-old male presented to the outpatient department with photophobia, ocular pain, and a sudden-onset, rapidly progressive diminution of vision in both eyes over two months duration. He denied any history of fever, weight loss, rashes, joint pains, backache, breathing difficulty, oral ulcers, burning micturition, or any h/o travel or exposure to pets.

At the presentation, his visual acuity in both eyes was the perception of light (PL+) with an inaccurate projection of rays. Intra-ocular pressure (IOP) was 27 and 24 mm of Hg, while the central corneal thickness (CCT) was 820 and 860 microns in the right (OD) and left eye (OS), respectively.

**Figure 1:**
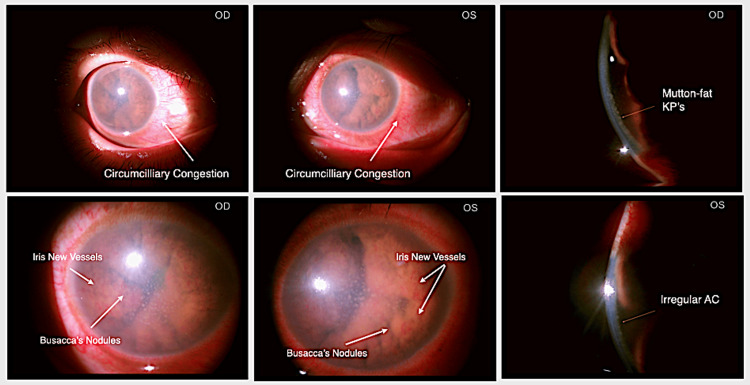
Anterior segment OU at presentation Slit lamp photograph showing OU: Circumciliary congestion, corneal edema, iris nodules with neovascularization, and shallow AC with mutton-fat KPs on the endothelium. Mutton-fat KP: Mutton-fat keratic precipitates; AC: Anterior chamber; OU: Both eyes

**Figure 2:**
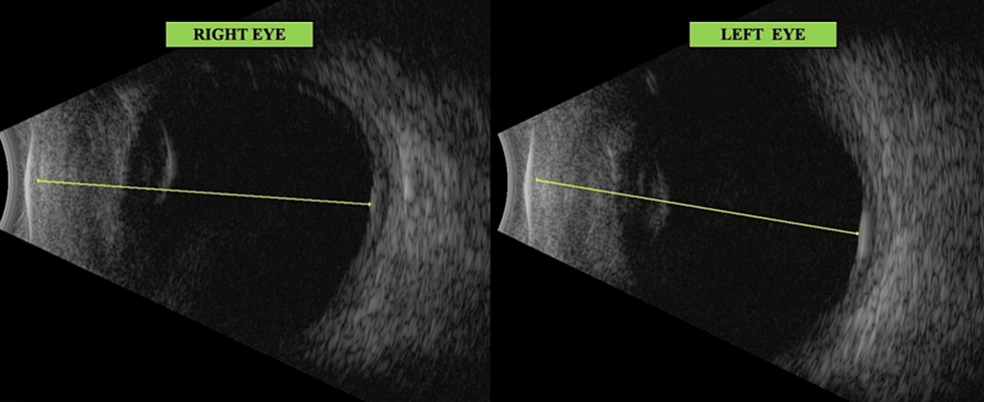
B-scan at presentation shows vitritis and choroidal thickening B-scan: Brightness scan

Slit lamp evaluation revealed circumciliary congestion, corneal oedema, granulomatous keratic precipitates, and a shallow anterior chamber (AC) with flare and grade 4 cells, while the iris showed neo-vascularisation with Busacca's nodules and seclusio pupillae. A complicated cataract prevented posterior segment visualization (Figure 1). Brightness scan (B scan) suggested bilateral vitritis and significant choroidal thickening (Figure 2).

Our working clinical diagnosis: Granulomatous panuveitis with mixed mechanism (angle closure with neovascular) glaucoma.

We considered the following differential diagnoses: Ocular tuberculosis, sarcoidosis, VKH, syphilis, and other vasculitic causes.

The patient was hospitalized and received intramuscular steroids, oral and topical IOP-lowering drugs, and an injection of sub-conjunctival steroid with mydricaine (modified: comprising 0.3 ml Atropine + 0.2 ml Adrenaline + 0.1 ml Lignocaine). 

**Figure 3:**
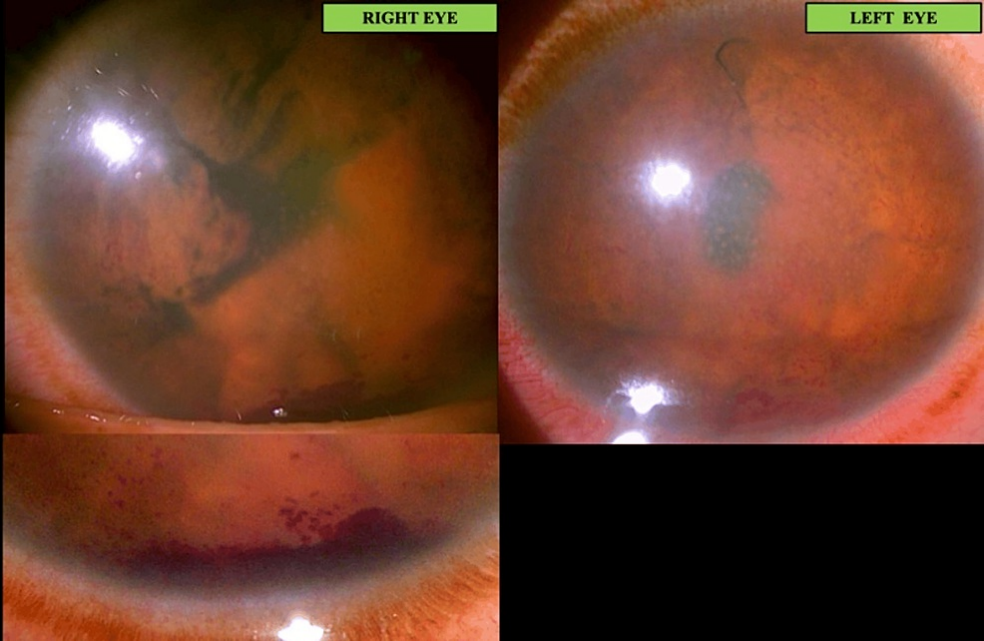
Hyphaema seen on day 2 Lowering of IOP due to anti-glaucoma drugs possibly led to decompression of the fragile new vessels of the rubeotic iris, causing hyphaema. Alternatively, it was due to worsening of the neovascularisation. Oedema, though slightly reduced, was still seen to persist. IOP: Intraocular pressure

A review of the patient on day two revealed a lowered IOP (10mmHg in both eyes), but this was found to precipitate hyphema from the rubeotic iris vessels (Figure 3). Definitive lab results were yet to be received.

This necessitated the administration of intravenous methylprednisolone (IVMP), which was given under the cover of anti-tubercular therapy (ATT) for presumed ocular TB under the supervision of the pulmonologist.

Investigations: Other than a borderline high CBC and erythrocyte sedimentation rate (ESR) (CBC-11,000, ESR-25, CRP-6) and positivity of antinuclear antibody (ANA), the other investigations were negative (Mantoux -Negative; Chest X-ray PA view- within normal limits (WNL); Serum calcium and angiotensin-converting enzyme (ACE) levels- WNL; Venereal Disease Research Laboratory Test for syphilis (VDRL)- Non-reactive; Urine routine microscopy- WNL; Rheumatoid (Arthritis) Factor (RA Factor) <10, C3 and C4 levels: normal).

Following an unsatisfactory response to three doses of IVMP, oral Azathioprine was added after clearance from the pulmonologist and physician. A posterior subtenon's triamcinolone injection was given.

**Figure 4:**
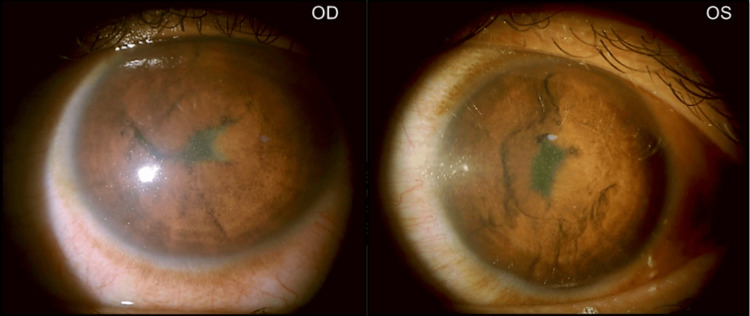
Anterior Segment after starting IVMP and azathioprine IVMP: Intravenous Methylprednisolone

**Figure 5:**
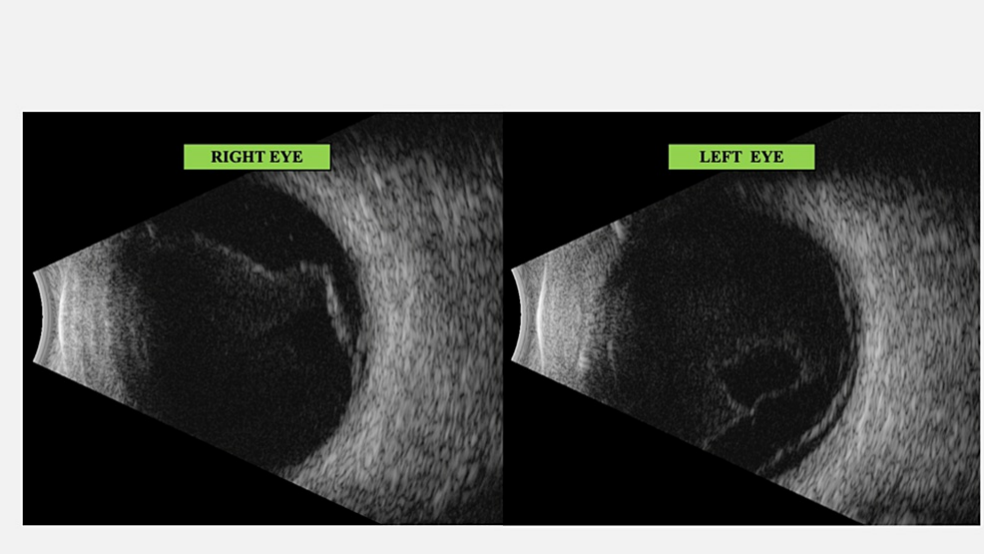
B-scan BE following immuno-suppressants The vitreous shows moderate low reflective dot echoes along with a membrane echo going from ONH to the periphery, disappearing on low gain suggestive of IPVD (incomplete Posterior vitreous detachment). The retina and optic nerve head appear within normal limits. Choroid thickening is seen, though reduced as compared to (Figure 2). ONH: Optic nerve head; B-scan: Brightness scan

Visual acuity improved bilaterally. The patient started perceiving hand movements (HM) with accurate projection in all quadrants. Corneal edema, keratic precipitates, anterior chamber cells, flare, rubeosis iridis, and hyphema were all reduced (Figure 4) after adding Azathioprine and having administered an additional fourth dose of IVMP. Even the B scan revealed reduced choroidal thickening (Figure 5).

At this point, at the patient's request, he was discharged with instructions on tapering oral steroids, continuing immunosuppression, topical steroids, and cycloplegics. He was advised to follow-up for review after one week.

**Figure 6:**
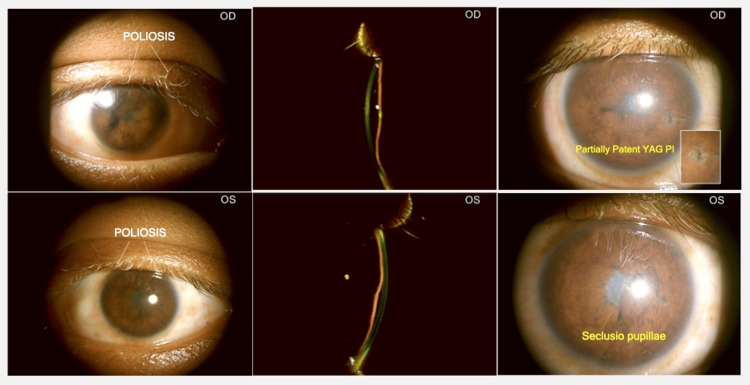
Anterior Segment: 1 month later: After 5 further doses of IVMP and OU LASER PI IVMP: Intravenous methylprednisolone; OU: Both eyes: PI: Peripheral iridotomy

**Figure 7:**
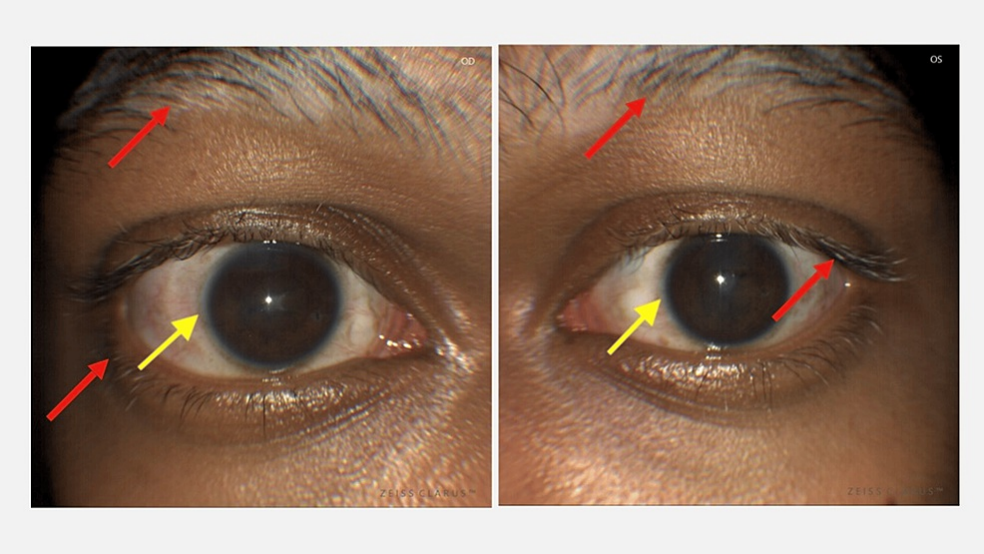
Signs of de-pigmentation at one month Poliosis of lashes and other dermatological changes (red arrows: vitiligo and alopecia of eyebrows) with Sugiura's sign (yellow arrows: peri-limbal vitiligo) seen one month after initial presentation

However, the patient was lost to follow-up and presented one month later after receiving five further doses of IVMP at a private hospital and having undergone a bilateral YAG PI (Figure 6). He was able to count fingers at 1 foot. Iris's new vessels had regressed, and IOP-lowering drugs were discontinued, but Azathioprine was continued. He presented now with poliosis of the eyebrows, eyelashes (Figure 7), and mustache, which were absent at the time of initial presentation.

A clinical confirmation of Vogt-Koyanagi-Harada syndrome was made. Due to the patient's poor socioeconomic status, more expensive definitive tests could not be performed. Definitive cataract surgery was planned under a guarded prognosis for visual rehabilitation with vitrectomy stand-by. However, the patient was lost to follow-up before being operated.

## Discussion

The mean age of presentation of VKH has been found to be between 32 to 50 years (range 20-65 years) across various reported case series with a strong female preponderance [4-9]. Our patient was a young male presenting at 20 years of age. The clinical presentation is usually bilateral [9], even though some unilateral presentations have been described [4]. Our patient also presented with bilateral ocular involvement.

Many case reports and case series suggest that posterior segment manifestations may be the initial presenting features of VKH. Posterior uveitis [4], serous or exudative retinal detachment, or disc oedema were likely presentations when the patient presented in the acute phase, while a depigmented sunset-glow fundus was the characteristic presentation of the chronic phase [8,9].

Moorthy et al. described VKH-related complications leading to visual loss. They found cataracts in 25%, glaucoma in 33%, and subretinal neovascular membranes (SRNVMs: late cause) in 10% [1]. Read described a series of patients where 51% developed at least one complication, which included cataracts in 42% of eyes, glaucoma in 27%, choroidal neovascular membranes in 11%, and subretinal fibrosis in 6% [7].

In a series by Arevalo et al, 54% of the VKH patients developed recurrences. All were treated with oral steroids. 33.8% developed glaucoma and 27.2% developed cataracts post-treatment [5], whereas Yoshitomi followed a series of 49 VKH patients over a minimum of 6 months and noted that 30% of them developed glaucoma over a median of 4.5 months duration; following pulse intravenous corticosteroid therapy [6].

In Lodhi's series [4], the incidence of glaucoma was 21.4% (88% drug-induced; more among acute VKH), and that of cataracts was 42.8% (more among chronic VKH). Seven percent developed choroidal neovascular membrane (CNVM), and 14% developed chronic vitritis. Secondary glaucoma developed in 22% of patients in the series by Alvarez-Guzman [10] and 11.7% in the series by Pandey et al., 15.8% being the overall glaucoma prevalence [11].

Eyes with glaucoma are more likely to present in the chronic recurrent stage of the disease [1,6,10]. Patients developing such complications had longer disease durations and recurrences [7]. Pre-treatment uveal effusion [11] and disc swelling were risk factors for glaucoma, whereas cataract progression and a worse final best-corrected visual acuity (BCVA) were post-treatment associations of glaucoma [6].

Glaucoma has been found to develop in cases of VKH through any of the following mechanisms:

Open-angle glaucoma: These constituted between 56-64% of all glaucomas presenting in VKH cases across series [11,12].

Angle-closure glaucoma: This constituted between 30%-44% [11,12] of glaucomas occurring in VKH cases. 82% of the secondary, post-treatment glaucomas seen by Alvarez-Guzman [10] had angle closure with the presence of anterior and posterior synechiae (in 86% and 76%, respectively), with iris bombe in 25%. 12% of all glaucomas in a series by Pandey et al. had acute angle closure crisis at presentation [11]. A case report by Yuan et al. describes bilateral acute angle closure glaucoma as the initial presentation in a patient with VKH [2].

Combined mechanism glaucomas: Pandey et al. reported these in 5.6% of the cases in their series [11].

Our patient presented with secondary angle closure due to seclusio pupillae (owing to granulomatous uveitis) and shallowing of the anterior chamber, corneal oedema, and high IOP. In addition, he also had a neovascular component with rubeosis iridis leading to hyphaema during his clinical course. Therefore, he had a combined mechanism of acute angle-closure/ neovascular glaucoma.

However, to the best of our knowledge, no VKH cases documented in literature hitherto have presented with (or have even developed) neovascular glaucoma like our case.

Forster et al. found that in 31% of those with glaucoma, medical therapy alone was sufficient to control intraocular pressure. The remaining 69% required some form of an invasive intervention, consisting of laser iridotomy, surgical iridectomy, trabeculectomy with or without 5-fluorouracil, and/or glaucoma drainage device implantation [12]. Pandey et al. found that the success of achieving IOP control is greater in those requiring medical/ laser treatment for glaucoma versus those where surgical management was required (64% versus 50% IOP control at 12 months) [11].

In our case, the initial management of glaucoma was in the form of oral and topical anti-glaucoma drugs. This reduced the intra-ocular pressure, but in two days, hyphaema was precipitated from the rubeotic iris vessels. The medication was reduced. Uveitis management with immunosuppressants led to regression of iris new vessels, and eventually, a peripheral iridectomy was done at 1 month with normalizing of IOP and discontinuation of all anti-glaucoma medication.

The clinical presentations of VKH have been divided into 4 phases [13]:

Prodromal

This phase spans 3-5 days and presents with auditory (tinnitus, hearing loss) or neurological manifestations (headache, meningismus). Lymphocytic pleocytosis is seen in cerebrospinal fluid (CSF) analysis.

Acute Uveitic Phase

This phase usually presents as bilateral blurring of vision. Diffuse posterior uveitis (choroiditis) occurs with peripapillary retinochoroidal thickening and optic disc swelling. Circumscribed retinal oedema with serous or exudative retinal detachments may be seen. Subretinal precipitates may also be present. Anterior chamber and vitreous inflammation may be seen. As per the series reported by Shreshta et al. [9] and Lodhi et al. [4], most patients with VKH presented in this phase. A better visual prognosis with aggressive treatment was seen in these cases [4].

Convalescence

The convalescent phase starts weeks after the uveitic phase and lasts several months. It presents with depigmentary skin changes (poliosis, vitiligo, alopecia), peri-limbal vitiligo (Sugiura's sign), and later, depigmentation of the choroid (sunset glow fundus).

Chronic Recurrent Phase

The chronic recurrent phase presents with panuveitis and acute exacerbations of anterior uveitis. Iris nodules occur, and vision-threatening complications develop (cataracts, glaucoma, subretinal neovascularization, etc.). A minority (25%) in Lodhi's case series [4] and a majority in Alvalez-Guzman's series [10] had chronic recurrent VKH at presentation.

Our patient presented with bilateral panuveitis and iris nodules with a mixed mechanism angle-closure/ neovascular glaucoma and a complicated cataract corresponding to the chronic phase with complications. It is rare for acute neovascular glaucoma to precede poliosis or to be a presenting feature in a case of VKH, as was seen in our case.

Early initiation of aggressive treatment with high-dose systemic steroids, especially IVMP, has been combined with peribulbar long-acting corticosteroids [4] and prolonged immunosuppression (with either corticosteroids alone at appropriate doses [9] or steroid-sparing immunosuppressants in a combination of both, where a steroid-sparing effect was achieved in 5-6 months with either mycophenolate mofetil or methotrexate) [14,15].

Such an aggressive strategy, especially when instituted in the acute phase, can help reduce recurrences, minimize complications, and improve visual prognosis [4,9]. A higher number of complications, a higher age at onset, and worse visual acuity at presentation are bad prognostic factors for final visual acuity [7]. Acute VKH at presentation and aggressive treatment have better visual prognosis [4,5] than chronic VKH.

Our patient received IVMP, sub-tenon's triamcinolone injection, and long-term Azathioprine for his uveitic process, in addition to a short treatment with anti-glaucoma medications followed by laser peripheral iridotomy for his secondary glaucoma. His vision improved from perception of light to counting fingers at half a meter. Cataract surgery was planned for further visual rehabilitation.

## Conclusions

VKH is a multi-systemic autoimmune disease usually affecting both eyes of middle-aged females. Our patient was a young adult male presenting with a mixed mechanism angle-closure/ neovascular glaucoma with bilateral panuveitis, iris nodules, and a complicated cataract. The clinician needs to be vigilant for signs of neovascular glaucoma as a rare presenting feature of VKH syndrome as in our case and hitherto unreported.

Detection of a case of VKH in its early stages may be challenging. Any treatment delay is likely to lead to sight-threatening complications owing to prolonged ocular inflammation. Thus, the role of early diagnosis, along with prompt initiation of proactive treatment, is imperative. The anterior and posterior segment ophthalmologists need to work in tandem. Often, aggressive management with high-dose systemic steroids and immunosuppressants might be necessary. A team approach involving the immunologist, the internist, and the dermatologist is desirable. The role of adequate counseling of the patient and his relatives regarding the course and prognosis is essential.
